# Dutch Translation and Validation of the Headache-Specific Locus of Control Scale (HSLC-DV)

**DOI:** 10.1155/2018/3046235

**Published:** 2018-05-02

**Authors:** Marceline C. Willekens, Don Postel, Martin D. M. Keesenberg, Robert Lindeboom

**Affiliations:** ^1^Health Center “het Wantveld”, Noordwijk, Netherlands; ^2^Corpus Mentis, Center for Physical Therapy & Science, Leiden, Netherlands; ^3^Academic Medical Center, Department of Clinical Epidemiology and Biostatistics, University of Amsterdam, Amsterdam, Netherlands

## Abstract

**Background and Objective:**

The assessment of locus of control forms an important part of headache treatment, and there is need to adapting them to the Dutch population.

**Methods:**

Forward-backward translation was used to obtain the Headache-Specific Locus of Control Scale–Dutch Version (HSLC-DV). The response of 87 participants with migraine, tension-type headache, and cervicogenic headache, aged between 18 and 55 years (75% female), is used. Test-retest reliability was measured by intraclass correlations. Construct validity was assessed by correlations with corresponding domains of the Pain Coping and Cognition List (PCCL) and by confirmation of known groups hypotheses. Structural validity was evaluated by factor analysis (principal axis factoring).

**Results:**

The intraclass correlations for the External, Internal, and Chance domains were 0.79, 0.89, and 0.73, respectively. Internal consistencies for domains exceeded 0.73 and were similar to those observed in the original study. Convergent correlations were as expected and three of the seven known groups hypotheses were confirmed. Structural validity was supported by results of the factor analysis that matched the proposed structure of the original instrument.

**Conclusions:**

The HSLC-DV is a valid and reliable questionnaire for measuring the locus of control.

## 1. Introduction

Migraine and tension-type headaches are highly prevalent and have a strong impact on society [1, 2]. Locus of Control (LoC) is related to the impact of headaches and chronic pain [3]. The extent patients believe they can control events affecting their pain is influenced by the degree of LoC [4, 5]. Individuals with internal LoC believe that events in their life derive primarily from their own actions. Individuals with External LoC attribute outcomes of events to external circumstances, for example, “the intervention of powerful others such as health-care professionals.” Chance LoC represents unordered forces such as fate and luck [6]. Cognitive-behavioral therapy (CBT) is a successful and most common psychological approach in treating pain and is effective in reducing disability and catastrophic reaction [2]. Positive outcomes have been detected also in patients with chronic daily headaches [2]. CBT helps to reduce pain by learning how to manage the LoC in headaches [2].

Influencing the level of LoC of patients with headache leads to faster and more sustained recovery from headaches [7]. The patient empowerment proceeds from the perspective that optimal outcomes of an intervention are achieved when patients become active participants in their treatment process [3, 7].

The Headache-Specific Locus of Control Scale (HSLC) by Martin en Holroyd [4] was developed in the US using the framework from Rotter's social learning theory [8]. The original US English version [4] of the HSLC showed promising psychometric properties. Three-week test-retest reliabilities of the subscales ranged between 0.72 and 0.78. Internal consistency reliabilities ranged between 0.84 and 0.88 [6]. Danish [5] and Spanish [9] versions are available. The Danish version was reliable and valid in a multiethnic sample from a tertiary care headache center.

To assess LoC in Dutch patients with headache, the Pain Coping and Cognition List (PCCL) is used. However, the PCCL includes two domains measuring more global internal and external pain management and was not specifically developed for patients with headache. Headache-specific instruments can be useful to screen patients on their Internal or External LoC before the treatment as it may affect the outcome [7]. We argue that there is a need for a specific instrument to measure LoC in patients with headache in Dutch. We developed a Dutch version of the HSLC (HSLC-DV). The aim of the present study is to investigate the reliability and validity of the HSLC-DV.

## 2. Methods

### 2.1. Translation Procedure

After obtaining permission from the developer (Professor Kenneth Holroyd, Ohio University), the original HSLC questionnaire was translated by a translation agency. Two translators independently translated the original US English version of the HSLC into Dutch. A third independent translator merged these two Dutch HSLC versions into the best suitable Dutch translation for each item. Subsequently, this Dutch version of the HSLC was backward translated into English by two other independent translators of the translation agency. These independent translators were unacquainted with the original US English version of the HSLC. Another independent translator translated again forward into Dutch.

A focus group consisting of four content experts with regard to the target group achieved consensus on comprehensibility and translation. Subsequently, 20 health-care professionals (10 psychologists and 10 general practitioners) evaluated the equivalence between the original and the translated version of the HSLC. After this assessment, the final Dutch version was composed, the HSLC-DV (Appendix).

### 2.2. Participants

Eligible participants were patients with a history of headaches visiting two referral centers (“Corpus Mentis, Center for Physical Therapy & Science” or “het Wantveld”) for treatment. Additionally, patients were recruited through the websites of the Dutch Association for Physical Therapy & Science (part of Corpus Mentis) and two patient support groups of the Dutch Migraine Association. The participants of the support groups were already diagnosed with migraine by visiting a physician in the past. Migraine patients were not recruited in the referral centers because they are not visiting a physiotherapist for their headache problems on a regular basis. Patients with migraine [4], cervicogenic headache [9], and tension-type headache [4], between the ages of 18 and 55 years old with headache complaints lasting longer than two months, were included. Thirty-five patients were allocated to the migraine group and 52 to the Tension-Type and Cervicogenic Headache group. All participants were native Dutch speakers and signed informed consent after receiving information about the purpose and procedure of the research. Exclusion criteria were stroke, TIA, CVA, dementia, pregnancy/menopause, medication overuse or cluster headache, tumors, and use of alcohol or special medication.

### 2.3. Assessment Instruments and Procedure

The participants included from Wantveld and Corpus Mentis were diagnosed by the physical therapist through history taking and physical examination, and after inclusion they filled out the survey. Patients completed the survey after inclusion (T0) and after three weeks (T1). The survey included the HSLC-DV (Appendix), the Pain Coping and Cognition List (PCCL [10]), and the numeric pain rating scale (NPRS [11]).

The HSLC [3] is a self-report questionnaire and contains 33 items with a Likert response scale ranging from 1 (strongly disagree) to 5 (strongly agree). The questionnaire has three subscales consisting of 11 items each: Internal LoC, External LoC, and Chance LoC. Each subscale score may range from 11 to 55 points. Lower scores reflect lower levels of the trait being measured by a subscale [12].

The PCCL [10] has 42 items and measures dealing with pain, LoC, and pain cognitions, within four subscales: Pain Coping (11 items), Catastrophizing (12 items), Internal Pain Management (11 items), and External Pain Management (8 items). It uses a 6-point Likert scale: 1 (totally disagree) to 6 (totally agree). The total scores and subscale scores may range from 1 to 6 points (total score divided by number of questions that were answered).

The NPRS [11] was used as a global measure of the experienced pain intensity and runs from 0 (no pain) to 10 (the worst pain you can imagine). The patient was instructed to circle the number that reflects the severity of the pain in the past week.

A Global Rating of Change (GRC) was included at T1 to inquire to what extent the headache complaints were changed at follow-up. A score of −5 indicates much worsened symptoms compared to the previous measurement, 0 indicates unchanged, and +5 indicates a maximum improvement compared to the previous measurement.

Patients were recruited from May 2014 to February 2015. Questions on demographic and clinical characteristics were included in the T0 survey. Patients provided self-reported information including headache intensity, headache episodes and headache duration, age and gender as well as work status, children, doctors' visits, sport, education level, civil status, and medication use (Table 1).

The survey was provided on paper and as an online version. Participants who did not respond to the request to fill out the questionnaires received a reminder by e-mail after 10 days. After two reminders, patients were excluded from the analysis.

### 2.4. Reliability

Reproducibility of the HSLC-DV domain scores was assessed by comparing the scores on T0 and T1 using intraclass correlations (ICCs), (consistency model, single measures). The reproducibility of the item scores was investigated using weighted kappas. Weighted kappas were estimated by calculating ICCs [13]. Random and systematic measurement error of the HSLC-DV was evaluated through the method of Bland & Altman that examines the magnitude of the mean score differences between T0 and T1 and by calculating limits of agreement (LoA) [14]. LoA were calculated as the mean sumscore differences between T0 and T1 ±1.96 × the standard deviation of the differences. LoA were calculated for the total sample and for the subsample with GRC scores between −1 and +1 who were considered unchanged in their headache severity at follow-up. Internal consistency was evaluated by Cronbach's alpha and item-rest correlations. Cronbach's alpha values for the subscales were compared to those of the original US version [4] study and with the Danish version [5]. Item-rest correlations of >0.30 were considered as adequate [15].

### 2.5. Construct Validity

Validity of the Dutch HSLC was evaluated by the correlations of HSLC-DV domains with corresponding domains of the PCCL. We regarded correlations between 0.1 and 0.3 as low, between 0.3 and 0.5 as medium, and between 0.5 and 0.7 as high [16]. We expected a medium/high correlation (*r* > 0.40) between the domains Internal LoC and External LoC of the HSLC-DV and PCCL and between the HSLC-DV Chance LoC and PCCL Catastrophizing (convergent correlations) [4, 9]. We expected low correlations between dissimilar domains of both instruments (divergent correlations). In addition, we also tested mean score differences between subgroups based on the following hypotheses (“known groups” validation):

1. We expected lower Internal LoC scores for the Tension-Type and Cervicogenic Headache group compared to the Migraine-Type group. Tension-type headache and cervicogenic headache are more often the results of stress and burnout complaints that are in turn associated with lower Internal LoC scores [17, 18].
2. We expected higher Internal scores for higher educated people compared to lower educated people. According to Pellino and Oberst, a higher educational level may indicate that the individual has more problem-solving ability or a higher level of self-efficacy in dealing with chronic pain [19].
3. We expected higher Internal LoC scores for men compared to woman. Men were found to be more inclined to believe that headache problems and headache relief are determined by their own actions or behaviors [19–21].
4. We expected higher Internal LoC scores for subjects who actively practiced sports or engaged daily in at least 30 minutes of moderate physical activity compared to those who did not. The positive influence of sports activity on the production of endorphins reduces the stress hormone cortisol [22]. Previous studies found that subjects who actively practice sports have higher scores on the Internal LoC than others [18].
5. We expected higher External LoC scores for subjects under (medical) headache treatment compared to no-headache treatment. Higher levels of medication use and preference for medical treatment were associated with External LoC in the original US study and the Spanish validation [4, 9].
6. We expected higher Chance LoC scores with more frequent headache days per month [4].
7. We expected higher Chance LoC scores with longer duration in days of headache episodes [4].

### 2.6. Structural Validity

Structural validity of the HSLC-DV was examined by a principal axis factoring analysis with orthogonal (Varimax) rotation in order to test the purported subscale structure of the HSLC-DV. A forced three-factor structure was used. Factor loadings > 0.40 for individual items were considered indicative of subscale domain membership. Kaiser–Meyer–Olkin test was used to examine whether the data are suited for factor analysis. KMO test values may range between 0 and 1. Values below 0.50 are deemed unacceptable. KMO measure of sampling adequacy was 0.69 indicating that the sample was large enough to conduct a principal component analysis.

### 2.7. Statistical Analysis

Numerical data were presented as mean (SD) or median (range) as appropriate. Differences between clinical subgroups were assessed with independent *t*-test or a one-way ANOVA in case of >2 groups or a nonparametric variant when assumptions of equal variances and normality were not met. All hypotheses were tested two-sided. In our analyses, *P* < 0.05 signified statistical significance. Differences in baseline and follow-up scores of respondents were tested with paired *t*-tests. Convergent and divergent (Pearson's) correlations between the HSLC and subscales of the PCCL were obtained by bootstrapping based on 1000 bootstrap samples.

There were no missing values on individual HSLC-DV items on T0. For the respondents on T1, one patient had 3 missing items, two had two missing items, and seven patients had one missing item. Little's MCAR test was not significant, and missing item values were imputed by expectation-maximization.

## 3. Results

**Figure 1:**
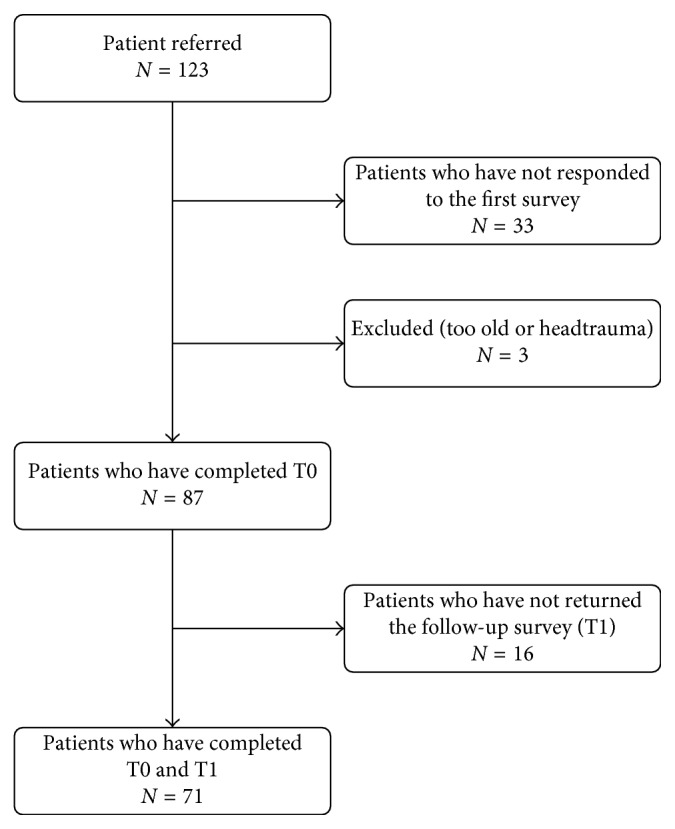
Flowchart.

A total of 87 patients with headache completed the survey at T0, and 16 patients did not return or completed the survey after three weeks at T1 (Figure 1). The baseline characteristics of the 16 patients that were lost to follow-up were not notably different. Baseline LoC subscale scores of the total sample were approximately normally distributed and were comparable to those of the patients who did not complete the study (Table 1). The online version was completed by 81 patients and six completed the paper version at T0. At T1, 6 patients completed the paper version and 65 patients completed the online version. The participants were outpatients from the referral centers (52 patients) or patient the support groups (35 patients).

### 3.1. Reliability of the HSLC-DV

The reproducibility and internal consistency results are summarized in Table 2. Cronbach's alpha ranged from 0.73 for Chance LoC to 0.89 for Internal LoC. These results are in line with the Danish study and the original US study. All item-rest correlations exceeded 0.30 except for two items of the External subscale and two items of the Chance subscale. Weighted kappas, as estimated using ICCs, for the items of the External LoC, Internal LoC, and Chance LoC ranged from 0.24 to 0.77 (median 0.58), 0.21 to 0.70 (median 0.59), and 0.43 to 0.68 (median 0.60), respectively.

The intraclass correlations (95%CI) for the External, Internal, and Chance domains were 0.77 (0.65–0.85), 0.81 (0.70–0.88), and 0.79 (0.67–0.86), respectively.

The mean difference between the External scores on the HSLC-DV between T0 and T1 was −0.42 points, and limits of agreement (LoA) were −11.2 to 10.4 points. The mean differences (LoA) for the Internal scores and the Chance scores were −0.94 (−12.1–10.2) and 0.85 (−8.7–10.4), respectively. For a subsample of the participants with GRC scores −1 to +1 (*N*=35), these were respectively 0.88 (−7.0–8.8), −0.96 (−11.7–9.8), and −0.97 (−9.0–7.1). Intraclass correlations in the subsample were practically equal to those calculated for the total sample: 0.83 (0.68–0.91), 0.82 (0.67–0.91), and 0.81 (0.66–0.90) for the External, Internal, and Chance scales, respectively.

### 3.2. Construct Validity of the HSLC-DV

Table 3 shows the correlations between HSLC-DV subscale scores and PCCL subscale scores. All convergent and divergent correlations were as hypothesized except for those between the HSLC Chance and PCCL Catastrophizing that had a correlation of 0.36 (*P* < 0.01). Pearson correlations between HSLC-DV subscales and PCCL pain or NPRS at T0 and T1 were generally small and not significant (*r* < 0.20, not shown in Table 3).

Table 4 shows the mean scores of clinical subgroups to test the construct validity hypotheses. From the seven HSLC hypotheses, three were confirmed (headache treatment, headache frequency, and headache duration) and four hypothesis were not confirmed (headache type, education, gender, and sports/moderate physical activity). For example, contrary to what was hypothesized, there was a higher mean score on Internal LoC for the Tension-Type and Cervicogenic Headache group compared with the Migraine group. Similarly, subjects who exercised at least 30 minutes of moderate physical activity or who actively engaged in sports had lower Internal LoC scores than those who did not (33.3 versus 38.7, *P*=0.01). Higher educated subjects had, as hypothesized, higher Internal scores (35.13 versus 34.65), however not significant (*P*=0.93).

### 3.3. Structural Validity of the HSLC-DV

The factor analysis (Table 5) largely confirmed the proposed three-factor structure of the HSLC-DV except for item 6 (Internal scale, low loading on all 3 factors) and items 18 (Chance item, loaded on External factor) and 29 (Chance item with low loading). The extracted factors explained 41% of the variance.

## 4. Discussion

We translated and validated the HSLC in a Dutch sample. The item reproducibility over a similar time interval as the original US study was generally good, except for item 4 (prevent headaches by not getting emotionally upset) and item 6 (prevent headaches by doctor). We found a comparable internal consistency as reported in the US and Danish validation studies. Most items contributed to the internal consistency although some items (items 6, 18, and 29) did not. The convergent correlations (>0.40) and divergent correlations (<0.30) between subscales of the HSLC-DV and related subscales of the PCCL were as expected. Only the HSLC Chance subscale correlated somewhat lower with the PCCL Catastrophizing scale (0.36). Catastrophizing in the US study was considered as a strategy for coping with headaches when chance or fate play a primary role in the onset of headache episodes. This is similar to what is measured by the HSLC Chance scale. Structural validity of the HSLC-DV was supported by the principal component analysis results. The vast majority of the items exclusively loaded on the intended subscales.

We expected a difference between men and woman. Men were found to be more inclined to believe that headache problems and headache relief are determined by their own actions or behaviors. This difference was not found in our results, comparable with Cano-García et al. [3].

From the seven HSLC known groups hypotheses, five were confirmed of which two reached no statistical significance. For example, contrary to what was expected, the mean Internal LoC score in the Tension-Type and Cervicogenic Headache group was higher than that in the Migraine-type group. We argue that the HSLC measures symptoms that may be present in all the three types of headaches. Hence, the hypotheses outcomes were not as expected for the different types.

Migraine patients are usually comorbid to depression [2]. The comorbidity with major affective disorders is more prevalent in subjects suffering from the chronic type of migraine, hence these patients report more frequently high levels of hopelessness and suicidal risk [2]. In our study, differences between chronic migraine and migraine were not investigated. Because we focused on the validation of the questionnaire, the factor depression was not processed in the analyses nor comorbidity factors in patients with headache.

No difference was observed for “sports or moderate physical activity for 30 min a day” with lower Internal LoC scores for physically active subjects.

### 4.1. Study Limitations

In our study, the relatively small sample size for the Principal Component Analysis is the first limitation. Further research in larger sample sizes should be completed to reach more definite conclusions regarding the structural validity of the HSLC-DV. A second limitation of this study is possible selection bias. A part of the sample may not be representative of the regular headache subjects visiting the referral centers for treatment because we also recruited patients from patient-support groups. This could have led to inclusion of patients with chronic headache with longer disease duration. We argue that patients with longer sustained headaches were more willing to participate in this study, as we can see in the high median duration of headaches in years at baseline of this group. In our study, more women (75%) were included than men. We argue that this could be a representative reflection of the headache population because women are affected 2 to 4 times more often by headaches than men [23]. A third limitation is the classification of headache types based on self-report. Patients of the support groups were not verified or seen by a physician and had to classify their type of headaches according to the given symptoms and definitions in the survey. The patient-support groups were associated with the Dutch Migraine Association and were approached for the inclusion of migraine patients, the most prevalent headache type. These participants were assumed confirmed migraine cases. We did not rule out possible secondary headaches in the migraine group. Despite this limitation, we argue that for the validation of this questionnaire, the importance is more on headache characteristics (localization, intensity, and duration) than on the underlying mechanism. The accuracy of the diagnosis is fundamental, and we want to promote more researches that could provide reliable results.

Finally, Nilsson and Bove have classified TTH and CH together as “musculoskeletal headache” [24]. We argue that the myogene headache group and migraine group represented a good heterogeneity for validation of the LoC questionnaire. For the validation of the HSLC, inclusion of these headache types better reflects the characteristics of the general population of patients with headache.

## 5. Conclusions

We conclude that the HSLC-DV is a reliable and valid instrument to measure LoC in a Dutch sample of patients with headache. Therefore, we strongly recommend the use of the HSLC-DV in the treatment counseling of patients with headache. Future research is necessary to determine cutoff points for the different scales of the HSLC-DV to identify patients with poor outcome on the headache treatment.

## Figures and Tables

**Table 1:** Baseline characteristics.

	Total sample (*N*=87)	Lost to follow-up (*N*=16)
Gender		
Male	21 (24%)	3 (19%)
Mean (SD) age in years	36 (9)	35 (7)
Headache type		
Migraine	35 (40%)	5 (31%)
Tension type and cervicogenic headache	52 (60%)	11 (69%)
Median (range) headache days per month	8 (1–31)	8 (4–30)
Median duration of headache in years	13 (1–45)	13 (3–30)
Median duration of headache period in days	3 (0.5–31)	2 (0.5–6)
Headache treatment	34 (39%)	4 (25%)
Work		
Full-time	40 (46%)	7 (44%)
Part-time	32 (37%)	7 (44%)
No paid work	11 (13%)	1 (6%)
Freelance	4 (4%)	1 (6%)
Median (range) number of children	0 (0–6)	1 (0–3)
Sports or moderate physical activity for 30 min a day^1^		
No sports or moderate physical activity	26 (30%)	9 (56%)
Moderate physical activity only	32 (37%)	4 (25%)
>2x a week sport	29 (33%)	3 (19%)
Education^2^		
Low	44 (51%)	8 (50%)
High	43 (49%)	8 (50%)
Civil status		
Single	18 (21%)	2 (13%)
Relation, living with partner, (re)married	65 (75%)	13 (81%)
Divorced	4 (5%)	1 (6%)
Medication		
Pain medication	63 (72%)	13 (81%)
Beta-blocker	2 (2%)	0 (0%)
Blood pressure-lowering drugs	3 (3%)	0 (0%)
Doctors visit in median times a year	2 (0–25)	2 (0–6)
HSLC		
External	24.2 (6.9)	23.4 (5.2)
Internal	34.9 (9.3)	37.8 (9.5)
Chance	33.8 (7.0)	35.0 (5.3)

Numbers are frequencies (%), mean (SD), or median (range). ^1^Dutch norm for healthy exercise. ^2^High education = higher vocational education and science education.

**Table 2:** Reproducibility and internal consistency of the HSLC-DV (*N*=87).

	Weighted kappa	Item-rest correlation	Cronbach's alpha if item deleted	Cronbach's alpha
External LoC				0.79 (0.75^∗^) (0.88^∗∗^)
HSLC6	0.24	0.12	0.80	
HSLC8	0.43	0.47	0.77	
HSLC10	0.44	0.56	0.76	
HSLC12	0.34	0.28	0.79	
HSLC14	0.61	0.54	0.76	
HSLC15	0.34	0.26	0.79	
HSLC16	0.69	0.55	0.76	
HSLC22	0.69	0.51	0.76	
HSLC24	0.77	0.47	0.77	
HSLC27	0.58	0.44	0.77	
HSLC30	0.62	0.61	0.75	

Internal LoC				0.89 (0.88^∗^) (0.86^∗∗^)
HSLC2	0.57	0.60	0.88	
HSLC4	0.21	0.56	0.88	
HSLC5	0.60	0.66	0.88	
HSLC7	0.59	0.52	0.89	
HSLC11	0.59	0.67	0.88	
HSLC17	0.68	0.79	0.87	
HSLC19	0.62	0.73	0.87	
HSLC21	0.53	0.61	0.88	
HSLC26	0.44	0.38	0.89	
HSLC28	0.70	0.67	0.88	
HSLC32	0.55	0.57	0.88	

Chance LoC				0.73 (0.71^∗^) (0.84^∗∗^)
HSLC1	0.60	0.49	0.70	
HSLC3	0.66	0.60	0.68	
HSLC9	0.63	0.43	0.71	
HSLC13	0.57	0.56	0.69	
HSLC18	0.58	−0.02	0.77	
HSLC20	0.43	0.32	0.72	
HSLC23	0.60	0.53	0.70	
HSLC25	0.61	0.55	0.69	
HSLC29	0.68	0.06	0.76	
HSLC31	0.51	0.39	0.71	
HSLC33	0.56	0.36	0.72	

LoC = Locus of Control subscale. Numbers correspond to the original HSLC scale. ^∗^Danish study. ^∗∗^Original US study.

**Table 3:** Pearson correlations between HSLC subscales and similar domains of the PCCL (*N*=87).

	PCCL external	PCCL internal	PCCL catastrophizing
HSLC external	**0.64** ^∗∗^	0.12	0.20
HSLC internal	−0.10	**0.42** ^∗∗^	−0.02
HSLC chance	0.27^∗^	−0.35^∗∗^	**0.36** ^∗∗^

Convergent correlations are given in bold. HSLC = Headache-Specific Locus of Control Scale; PCCL = Pain Coping and Cognition List. ^∗^ *P* < 0.05.  ^∗∗^ *P* < 0.01.

**Table 4:** Known groups and hypotheses (*N*=87).

		External	Internal	Chance
(1)	Migraine (*N*=35)	24.98 (7.20)	32.88 (9.78)	33.68 (6.68)
	Tension type + cervicogenic headache (*N*=52)	23.61 (6.65)	36.24 (8.87)	33.93 (7.25)
		*P*=0.34	**P** **=0** **.15**	*P*=0.76

(2)	Education, low (*N*=44)	25.47 (6.87)	34.65 (9.52)	34.36 (6.90)
	Education, high (*N*=43)	22.82 (6.67)	35.13 (9.26)	33.29 (7.12)
		*P*=0.08	**P** **=0** **.93**	*P*=0.37

(3)	Male (*N*=21)	23.97 (6.27)	37.70 (7.04)	32.34 (8.05)
	Female (*N*=66)	24.22 (7.09)	33.99 (9.84)	34.30 (6.61)
		*P*=0.89	**P** **=0** **.40**	*P*=0.30

(4)	No sport (*N*=26)	23.30 (5.65)	38.71 (9.74)	32.68 (5.92)
	Moderate activity + 2x wk sports (*N*=61)	24.53 (7.34)	33.26 (8.75)	34.32 (7.39)
		0.47	**P** **=0** **.0** **1** ^**∗**^	*P*=0.19

(5)	No treatment (*N*=53)	22.99 (6.15)	35.39 (9.97)	33.54 (6.44)
	Treatment (*N*=34)	25.99 (7.59)	34.11 (8.36)	34.27 (7.85)
		**P** **=0** **.0** **4** ^**∗**^	*P*=0.45	*P*=0.43

(6)	0–8 headache days per month (*N*=44)	23.73 (6.01)	35.76 (8.34)	31.22 (6.35)
	9–31 headache days per month (*N*=43)	24.60 (7.69)	34.00 (10.29)	36.50 (6.65)
		*P*=0.54	*P*=0.92	**P<** **0** **.00** **1** ^**∗**^

(7)	0–3 days per headache period (*N*=58)	24.14 (6.1)	34.84 (8.89)	32.54 (6.46)
	4–31 days per headache period (*N*=29)	24.20 (8.30)	36.75 (9.05)	36.40 (7.40)
		*P*=0.99	*P*=0.56	**P** **=0** **.0** **1** ^**∗**^

*P* values for construct validity hypotheses are given in bold. HSLC = Headache-Specific Locus of Control Scale. Scores are mean (SD). Score differences are tested using the Mann–Whitney *U* test. ^∗^ *P* < 0.05.

**Table 5:** Structural validity: factor loadings on the purported subscales of the HSLC-DV scale.

		Internal LoC eigenvalue = 6.8	External LoC eigenvalue = 4.3	Chance LoC eigenvalue = 2.4
External LoC	HSLC6^∗^	0.168	**0.100**	0.228
	HSLC8	0.190	**0.512**	0.229
	HSLC10	0.230	**0.687**	−0.080
	HSLC12	0.056	**0.336**	0.118
	HSLC14	−0.108	**0.643**	0.153
	HSLC15^∗^	−0.093	**0.356**	0.145
	HSLC16	−0.112	**0.720**	0.028
	HSLC22	0.220	**0.619**	−0.134
	HSLC24	−0.052	**0.639**	0.188
	HSLC27	0.030	**0.534**	0.037
	HSLC30	0.062	**0.702**	0.057

Internal LoC	HSLC2	**0.660**	−0.013	−0.123
	HSLC4	**0.596**	0.037	−0.157
	HSLC5	**0.699**	0.000	−0.195
	HSLC7	**0.638**	0.147	0.023
	HSLC11	**0.729**	−0.012	−0.076
	HSLC17	**0.796**	0.061	−0.216
	HSLC19	**0.845**	0.054	0.037
	HSLC21	**0.600**	0.084	−0.353
	HSLC26	**0.429**	−0.067	−0.281
	HSLC28	**0.742**	−0.041	−0.083
	HSLC32	**0.618**	0.019	−0.201

Chance LoC	HSLC1	−0.224	−0.148	**0.650**
	HSLC3	−0.205	−0.217	**0.707**
	HSLC9	−0.249	0.019	**0.568**
	HSLC13	−0.142	0.122	**0.650**
	HSLC18^∗^	0.479	0.114	**0.096**
	HSLC20	0.041	0.162	**0.423**
	HSLC23	−0.020	0.186	**0.694**
	HSLC25	−0.129	0.271	**0.674**
	HSLC29^∗^	−0.032	−0.223	**0.088**
	HSLC31	−0.130	0.163	**0.484**
	HSLC33	−0.276	−0.181	**0.415**

Three factors explained 41% of the total variance in HSLC-DV scores. ^∗^Cross-loadings or items that do not load (<0.40) on the purported subscale.

**Table 6 tab6:** Hoofdpijn-Specifiek Locus of Control (HSLC-DV) scale

1.	Als ik hoofdpijn heb is er niets wat ik kan doen om het beloop te veranderen	1	2	3	4	5
2.	Ik ben in staat een deel van mijn hoofdpijn te voorkomen door het vermijden van bepaalde stressvolle situaties	1	2	3	4	5
3.	Ik ben compleet machteloos met betrekking tot mijn hoofdpijn	1	2	3	4	5
4.	Ik kan hoofdpijn soms voorkomen door niet overstuur te raken	1	2	3	4	5
5.	Wanneer ik zorg voor voldoende rust heb ik minder vaak hoofdpijn	1	2	3	4	5
6.	Alleen mijn arts kan mij aanwijzingen geven om mijn hoofdpijn te voorkomen	1	2	3	4	5
7.	Mijn hoofdpijn is soms erger omdat ik overactief ben	1	2	3	4	5
8.	Mijn hoofdpijn kan minder erg zijn wanneer medische professionals mij goede zorg verlenen. (Artsen, zusters, etc.)	1	2	3	4	5
9.	Ik heb geen enkele invloed op mijn hoofdpijn	1	2	3	4	5
10.	De behandeling van mijn arts kan mij helpen tegen hoofdpijn	1	2	3	4	5
11.	Wanneer ik mij zorgen maak of pieker over iets heb ik een grotere kans op hoofdpijn	1	2	3	4	5
12.	Alleen al een bezoek aan mijn arts helpt tegen mijn hoofdpijn	1	2	3	4	5
13.	Ongeacht wat ik doe: als ik hoofdpijn zal krijgen, dan krijg ik het ook	1	2	3	4	5
14.	Regelmatig contact met mijn arts is de beste manier voor mij om controle te krijgen over mijn hoofdpijn	1	2	3	4	5
15.	Wanneer ik hoofdpijn heb dien ik een medische deskundige te raadplegen	1	2	3	4	5
16.	Het zorgvuldig volgen van de door mijn arts uitgeschreven medicijnenkuur is de beste manier om hoofdpijn te voorkomen	1	2	3	4	5
17.	Wanneer ik teveel van mijzelf vraag krijg ik hoofdpijn	1	2	3	4	5
18.	Geluk speelt een grote rol bij het bepalen hoe snel ik zal herstellen van hoofdpijn	1	2	3	4	5
19.	Door er voor te zorgen dat ik niet overactief of geïrriteerd raak voorkom ik veel hoofdpijn	1	2	3	4	5
20.	Het niet krijgen van hoofdpijn is voornamelijk een kwestie van geluk	1	2	3	4	5
21.	De dingen die ik doe beïnvloeden de kans op hoofdpijn	1	2	3	4	5
22.	Gewoonlijk herstel ik van een hoofdpijn na het ontvangen van goede medische zorg	1	2	3	4	5
23.	Ik heb een grote kans op hoofdpijn, ongeacht wat ik doe	1	2	3	4	5
24.	Wanneer ik niet de juiste medicatie heb, heb ik last van hoofdpijn	1	2	3	4	5
25.	Vaak heb ik het gevoel dat wat ik ook doe ik toch hoofdpijn zal krijgen	1	2	3	4	5
26.	Ik ben zelf verantwoordelijk voor het krijgen van hoofdpijn	1	2	3	4	5
27.	Wanneer mijn arts een vergissing maakt, ben ik degene die daaronder lijdt door hoofdpijn	1	2	3	4	5
28.	Mijn hoofdpijn wordt erger wanneer ik met stress te maken heb	1	2	3	4	5
29.	Wanneer ik hoofdpijn krijg moet ik de natuur gewoon zijn gang laten gaan	1	2	3	4	5
30.	Professionele medische deskundigen zorgen dat ik geen hoofdpijn krijg	1	2	3	4	5
31.	Ik heb simpelweg geluk wanneer ik een maand geen hoofdpijn heb	1	2	3	4	5
32.	Wanneer ik niet goed voor mezelf zorg heb ik een grote kans op hoofdpijn	1	2	3	4	5
33.	Het is een kwestie van toeval of ik hoofdpijn krijg	1	2	3	4	5

## Appendix

### Hoofdpijn-Specifiek Locus of Control (HSLC-DV)

*Instructies*: Deze vragenlijst geeft inzicht in de klachten die u ervaart door de hoofdpijn. Hieronder staan 33 stellingen waarmee u het eens of oneens bent. Naast iedere stelling staan de getallen 1 tot en met 5. Hiermee geeft u aan in welke mate u het eens of oneens bent met de stelling. De 1 staat voor “Volkomen oneens” en de 5 staat voor “Volkomen eens.” Omcirkel het getal dat correspondeert met de mate waarin u het eens of oneens bent met de stelling. Zorg ervoor dat u achter *alle 33 stellingen* een getal heeft omcirkeld. *Slechts één getal* per stelling kan worden omcirkeld. De antwoorden die u geeft zijn uw persoonlijk mening. Er zijn dan ook geen goede of foute antwoorden mogelijk. [Table tab6]

  1 = Volkomen oneens
  2 = Gematigd oneens
  3 = Neutraal
  4 = Gematigd eens
  5 = Volkomen eens

Explanation domains:

- External subscale: 6, 8, 10, 12, 14, 15, 16, 22, 24, 27, 30.
- Internal subscale: 2, 4, 5, 7, 11, 17, 19, 21, 26, 28, 32.
- Chance subscale: 1, 3, 9, 13, 18, 20, 23, 25, 29, 31, 33.
